# Evidence-Based Human Homeopathy and Veterinary Homeopathy. Comment on Bergh et al. A Systematic Review of Complementary and Alternative Veterinary Medicine: “Miscellaneous Therapies”. *Animals* 2021, *11*, 3356

**DOI:** 10.3390/ani12162097

**Published:** 2022-08-17

**Authors:** Petra Weiermayer, Michael Frass, Thomas Peinbauer, Liesbeth Ellinger, Edward De Beukelaer

**Affiliations:** 1 Veterinary Practice Dr. Weiermayer, 1130 Vienna, Austria; 2 Scientific Society for Homeopathy, 06366 Koethen, Germany; 3 Medical Faculty, Johannes Kepler University, 4040 Linz, Austria; 4 Centaurea, 7312 Apeldoorn, The Netherlands; 5 Riverside Veterinary Centre, Marlborough SN8 2AG, UK

**Keywords:** evidence, homeopathy, veterinary homeopathy, One Health, antimicrobial resistance

## Abstract

**Simple Summary:**

According to the simile principle (*Similia similibus curentur,* or ‘Let like be cured by like’), classical (=individualized) homeopathic therapy is based on the individual and not on the indication. Based on the following three facts, the discussion of the evidence in human and veterinary homeopathy lays the foundations for a comprehensive presentation of the evidence on homeopathy: (a) homeopathic medicinal products without indication are 100% identical with regard to production, quality, safety, and principles of application, regardless of whether they are used in animals or humans; (b) if the simile principle is adhered to that classical (=individualized) homeopathic therapy is based on the individual and not on the indication; and (c) if the proof of effectiveness of individualized homeopathy in one or more indications is available, the logical consequence seems to be that it can be concluded that it is effective in other indications. When criteria for evidence-based medicine for design, conduction, documentation, and rating of studies in homeopathy are considered, effects on all quality levels according to Cochrane criteria are recognizable, especially for individualized homeopathy, even in methodologically high-quality studies. In view of One Health and of the demands of the European Green Deal (Farm2Fork Strategy) and the EU Organic Regulation 2018/848, the application of homeopathy in the sense of integrative veterinary medicine (a combination of patient-oriented conventional and complementary veterinary medicine) and the integration of complementary medicine including homeopathy at the universities seems a necessary consequence and requirement in the interests of the patient.

**Abstract:**

(1) Background: Classical (=individualized) homeopathic therapy is based on the individual and not on the indication. (2) Methods: The prerequisite for conducting methodologically high-quality studies on indvidualized homeopathy is that the principles of homeopathy are considered, since the selection of the simile (the individually appropriate homeopathic medicinal product) is decisive for the effectiveness of the homeopathic treatment, because only an application lege artis can be effective for the respective patient. Apart from this, criteria for evidence-based medicine must be considered for design, conduction, documentation, and rating of studies in homeopathy. (3) Results: When criteria for evidence-based medicine for design, conduction, documentation, and rating of studies in homeopathy are considered, effects on all quality levels according to Cochrane criteria are recognizable, especially for individualized homeopathy, even in the methodologically high-quality studies. (4) Conclusions: Based on the following three facts, the discussion of the evidence in human and veterinary homeopathy lays the foundations for a comprehensive presentation of the evidence on homeopathy: (a) homeopathic medicinal products without indication are 100% identical with regard to production, quality, safety, and principles of application, regardless of whether they are used in animals or humans; (b) if the simile principle (*Similia similibus curentur,* or ‘Let like be cured by like’) is adhered to that classical (=individualized) homeopathic therapy is based on the individual and not on the indication; and (c) if the proof of effectiveness of individualized homeopathy in one or more indications is available, the logical consequence seems to be that it can be concluded that it is effective in other indications. In view of One Health and of the demands of the European Green Deal (Farm2Fork Strategy) and the EU Organic Regulation 2018/848, the application of homeopathy in the sense of integrative veterinary medicine and the integration of complementary medicine including homeopathy at universities seems a necessary consequence and requirement in the interests of the patient, which is already expressed in the American consensus guidelines for an integrative veterinary medicine curriculum and is legally anchored in Switzerland by the Medical Professions Act for university teaching and research.

## 1. Introduction

The way homeopathy has been represented over the past years as well as in the article ‘*A Systematic Review of Complementary and Alternative Veterinary Medicine: “Miscellaneous Therapies”*’, a systematic review on published papers [1], clearly shows the need of in-depth knowledge of integrative medicine (a combination of patient-oriented conventional and complementary medicine). In the US, integrative medicine is a self-evident part of the curriculum of the veterinary schools and of the top medical schools, from Harvard to Stanford. In Europe, there is hardly any academic integration and very few public or university funds available for research into integrative medicine, with the exception of Switzerland. Especially, in view of the global threat posed by antibiotic resistance, the integration of complementary medicine including homeopathy at universities is a necessary consequence and requirement in the interests of the patient. Fortunately, it is already expressed in the American consensus guidelines for an integrative veterinary medicine curriculum and is legally anchored in Switzerland by the Medical Professions Act for university teaching and research [2,3,4].

The German physician Dr. Samuel Hahnemann (1755–1843) developed the medical system of individualized, so-called classical homeopathy or single-substance homeopathy. The treatment is based on the law of similars—*Similia similibus curentur*, or ‘Let like be cured by like’. The patient’s individual symptoms lead to the simile, i.e., the homeopathic medicinal product, the symptoms of which, generated in healthy individuals, best reflect the patient’s symptoms [5]. In accordance with the regulations of the European Pharmacopoeia or of the Homeopathic Pharmacopoeia, homeopathic medicinal products are produced in a standardized manner [6,7].

## 2. Materials and Methods

Since the selection of the simile is decisive for the effectiveness of the homeopathic treatment, for conducting methodologically high-quality studies on individualized homeopathy it is the prerequisite that the principles of homeopathy are considered. Only an application lege artis can be effective for the respective patient. It is unlikely that the desired effectiveness will follow, if the individually appropriate homeopathic medicinal product, the simile, is not selected by appropriately trained and experienced homeopathic doctors/veterinarians according to the basic homeopathic principles [4]. A study of 2008 already confirmed that, for the successful application of individualized homeopathy, the basic principles—i.e., individualized selection of a homeopathic medicinal product according to the principle of similarity—must be taken into account [8]. Research in individualized homeopathy that does not respect the essential basic principles of individualized homeopathy prescribing will inevitably lead to negative study outcomes [4,9]. Apart from this, criteria for evidence-based medicine must be considered for design, conduction, documentation, and rating of studies in homeopathy.

## 3. Results

Up to 2014, five of the six meta-analyses on various indications concluded that the effectiveness of homeopathic therapy differs from that of placebo [10,11,12,13,14]. Of a total of 131 original articles, 13 RCTs with minimal risk of bias were identified in the review program from 2014, 2017, 2018, and 2019 [14,15,16,17]. Ten of these RCTs testing homeopathy in comparison to placebo resulted in a mean OR of 1.68 (CI = 1.25–2.24; *p* < 0.001), i.e., a statistical significance for the effectiveness of homeopathy compared with placebo. Such ‘effect size’ seems comparable with, for example, sumatriptan for migraine, fluoxetine for major depressive disorder, and cholinesterase inhibitors for dementia [14]. Five of the 13 RCTs with minimal risk of bias also showed high reliable evidence [18,19,20,21,22]. Especially for individualized homeopathy, effects on all quality levels according to Cochrane criteria are recognizable, even in the methodologically high-quality studies, when criteria for evidence-based medicine for design, conduction, documentation, and rating of studies in homeopathy are applied, e.g., all high-quality trials on homeopathy are considered for rating [4]. As the authors of the article ‘*A Systematic Review of Complementary and Alternative Veterinary Medicine: “Miscellaneous Therapies”*’ exclude farm animals from the systematic review, they exclude 16 out of 18 RCTs on veterinary homeopathy which were analyzed by the meta-analysis of 2015 [23]. However, further high-quality studies are also necessary in human and veterinary homeopathy, as in the majority of the fields of human and veterinary medicine. For veterinary homeopathy, the review of 2014 and the meta-analysis of 2015 showed evidence of the effectiveness of veterinary homeopathy compared to placebo (*p* = 0.01 for n = 15, pooled OR = 1.69 (CI = 1.12–2.56), *p* = 0.02 for n = 2, pooled OR = 2.62 (CI = 1.13–6.05)) [23,24]. In addition to studies to demonstrate the effectiveness of homeopathy in infections, data from health care research, so-called ‘real world data’, show the potential for a significant reduction in the use of antibiotics through homeopathic treatments [4].

## 4. Discussion

A 2007 Cochrane review of systematic reviews of predominantly conventional therapies revealed that 96% of all systematic reviews call for more methodologically high-quality research [25]. Forty-nine percent of these publications present results that do not allow any conclusions to be drawn about the benefit/harmfulness of the examined intervention. According to this review, 7% of all medical procedures are actually harmful. Only 1.38% of conventional therapies are definitely effective, 43% are classed as effective, but the studies show methodological deficiencies. Based on the results of the meta-analyses of 2014, 2015, 2017, 2018, and 2019, homeopathy should be classified provisionally in the group of therapies (conventional 44%: 1.38% plus 43%), which are effective but need further research [14,15,16,17,25]. When criteria for evidence-based medicine for design, conduction, documentation, and rating of studies in homeopathy are considered, e.g., all high-quality trials on homeopathy are considered for rating, especially for individualized homeopathy, effects on all quality levels according to Cochrane criteria are recognizable, even in the methodologically high-quality studies [4]. A review of 2013 already confirmed that more than 90% of all studies had to be excluded in order to be able to conclude that homeopathy is not effective, a common practice applied in some reviews and meta-analyses on homeopathy with negative outcome [4,26].

By definition, modern evidence-based medicine (EBM) is based on three pillars: the current state of scientific research, the clinical experience of doctors and veterinarians, and the values and wishes of clients and patients [27]. Homeopathy is based on all three pillars of modern evidence-based medicine. There seems to be a confusion in the article ‘*A Systematic Review of Complementary and Alternative Veterinary Medicine: “Miscellaneous Therapies”*’, which describes the different results and outcome variables, that for an intervention to be considered as being effective it needs to be able to be ‘explainable’. In other words, ‘scientific’ can only be associated with explanations of the mode of action of treatments based on the current anatomopathological approach to medicine and that only medical techniques based on these models deserve attention from the research community. However, knowing how a medicine works has never been intended to be a prerequisite for its use according to the founder of modern evidence-based medicine, David Sackett [27]. Aspirin (acetylsalicylic acid) is one of the most widely used drugs in the world, yet it was used for over 70 years before its mechanism of action was discovered in 1971 [28]. Science is a constantly evolving field and what the scientific establishment declares to be ‘impossible’ in one era, is often proved to be ‘fact’ in another [29].

In view of One Health and of the demands of the European Green Deal (Farm2Fork Strategy) and the EU Organic Regulation 2018/848:

(1) to reduce the use of antibiotics by 50% throughout the EU by 2030
(2) to increase the number of organic farms in the EU from 8% to 25% by 2030
(3) to give preference to homeopathy and phytotherapy in organic farms before conventional medicines are used, including antibiotics, the application of these complementary medical disciplines in the sense of integrative veterinary medicine, i.e., to combine best practices of conventional and complementary medical therapy procedures, seems proactive and innovative [30,31]. Hence, the integration of complementary medicine including homeopathy at the universities seems a necessary consequence and requirement in the interests of the patient.

## 5. Conclusions

Evidence for the effectiveness of human and veterinary homeopathy in general, and in particular in the treatment of infections, has been sufficiently proven to justify further research in homeopathy [32]. Hence, obvious non-scientific interests might consequently have led to misinformation about homeopathy in general and within the article ‘*A Systematic Review of Complementary and Alternative Veterinary Medicine: “Miscellaneous Therapies”*’ [33]. Based on the following three facts, the discussion of the evidence in human and veterinary homeopathy lays the foundations for a comprehensive presentation of the evidence on homeopathy: (a) homeopathic medicinal products without indication are 100% identical with regard to production, quality, safety, and principles of application, regardless of whether they are used in animals or humans; (b) if the simile principle (*Similia similibus curentur*, or ‘Let like be cured by like’) is adhered to that classical (=individualized) homeopathic therapy is based on the individual and not on the indication; and (c) if the proof of effectiveness of individualized homeopathy in one or more indications is available, the logical consequence seems to be that it can be concluded that it is effective in other indications [34]. Especially for individualized homeopathy, effects on all quality levels according to Cochrane criteria are recognizable, even in the methodologically high-quality studies, when criteria for evidence-based medicine for design, conduction, documentation, and rating of studies in homeopathy are considered, e.g., all high-quality trials on homeopathy are considered for rating [4]. In view of One Health and of the demands of the European Green Deal (Farm2Fork Strategy) and the EU Organic Regulation 2018/848, the application of homeopathy in the sense of integrative veterinary medicine and the integration of complementary medicine including homeopathy at universities seems a necessary consequence and requirement in the interests of the patient, which is already expressed in the American consensus guidelines for an integrative veterinary medicine curriculum and is legally anchored in Switzerland by the Medical Professions Act for university teaching and research [2,3,30,31]. 
